# Establishment and validation of a phenotype-driven predictive model for the diagnostic efficacy of trio-based whole exome sequencing (trio-WES) in children with genetic neurodevelopmental disorders (g-NDDs)

**DOI:** 10.3389/fneur.2025.1574021

**Published:** 2025-10-15

**Authors:** Ruohao Wu, Xiangyang Luo, Zhe Meng, Wenting Tang, Liyang Liang

**Affiliations:** ^1^Department of Children’s Medical Center, Sun Yat-sen Memorial Hospital, Sun Yat-sen University, Guangzhou, Guangdong, China; ^2^Weierkang Children’s Rehabilitation Center, Guangzhou, Guangdong, China; ^3^Department of Research and Molecular Diagnostics, Sun Yat-sen University Cancer Center, Sun Yat-sen University, Guangzhou, Guangdong, China

**Keywords:** genetic neurodevelopmental disorders, neurodevelopmental comorbidity, trio-based whole-exome sequencing, exon-level variants, diagnostic efficacy, phenotype-driven, alignment diagram

## Abstract

**Background:**

Genetic neurodevelopmental disorders (g-NDDs), a complex group of idiopathic syndromes with a heterogeneous genetic etiology, are defined as global developmental delay/intellectual disability (GDD/ID) with other common neurodevelopmental comorbidity (NDC), such as autism spectrum disorder (ASD). Although significant progress has been made in trio-based whole-exome sequencing (trio-WES) that enable the detection of exon-level variants, the diagnostic efficacy of using trio-WES for g-NDDs is still not satisfactory. Therefore, exploring key phenotypic variables for forecasting the diagnostic probability of trio-WES is extremely necessary to implement personalized diagnosis for children with g-NDDs.

**Methods:**

A total of 265 g-NDDs children who received trio-WES at Sun Yat-sen Memorial Hospital between Sep 2016 and Dec 2022 were enrolled and clustered temporally into training and internal validation sets [163 cases (Oct 2016 ~ Dec 2022) and 102 cases (Sep 2016 ~ Sep 2018), respectively]. A total of 97 g-NDDs children who underwent trio-WES at Weierkang Children’s Rehabilitation Center between Jan 2023 and Dec 2023 were enrolled and served as an independent external validation set. Univariate and multivariate logistic regression were conducted in the training set to screen out independent diagnosis-related phenotypic signifiers and establish an alignment diagram model. The model was further validated in internal and external validation sets.

**Results:**

Through univariate and multivariate analyses, independent diagnosis-related predictive signifiers, including GDD/ID severity, NDC complexity, ASD, and head circumference abnormality, were identified in the training cohort and used to construct a model. The model showed good discrimination power with an area under the ROC curves (AUC) in the training set of 0.821 (95% CI: 0.756–0.886), yielding a F1 score of 0.76. The model also showed powerful prediction in both the internal (AUC: 0.905 with 95% CI: 0.842–0.968 and F1 score: 0.77) and external validation sets (AUC: 0.919 with 95% CI: 0.858–0.979 and F1 score: 0.79).

**Conclusion:**

We found the potential linear relationship between trio-WES-diagnostic rates and the phenotypic enrichments in g-NDDs patients for the first time, indicating the possibility of applying a logistic regression model based on phenotypic features to predict the personalized diagnostic rates of using trio-WES in children with g-NDDs.

## Introduction

Genetic neurodevelopmental disorders (g-NDDs), a complex group of idiopathic syndromes that tends to have a heterogeneous genetic etiology and represents a complex group of neurological diseases with marked clinical variability and readily observable deficits from a very early age (often <6 months), affecting approximately 1% ~ 3% of children worldwide and exerting a substantial burden on society and patients’ families (1). They mainly comprise global developmental delay/intellectual disability (GDD/ID), autism spectrum disorder (ASD), attention deficit hyperactivity disorder (ADHD), and epilepsy (EP) (2). Among these neurological disease conditions, GDD/ID is the major and essential part of g-NDDs (3); while other common neurological disease conditions, such as ASD, ADHD, and EP, can present as neurodevelopmental comorbidity (NDC), solely or jointly appearing in individuals with g-NDDs (4). Therapeutic intervention/treatment or disease management for g-NDDs is individualized, multidisciplinary, and mainly symptomatic treatment, such as rehabilitation therapy and pharmacotherapy (like encephalon glycoside and exogenous nerve growth factor).

Genetic alterations are considered to play a key role in the pathogenesis of g-NDDs. For instance, 30–50% of g-NDDs cases were reported to be caused by single-nucleotide variants (SNVs), copy-number variants (CNVs) and chromosomal abnormalities; Rett, fragile-X, and Down syndromes are considered as the three most common types of g-NDDs worldwide (5). Owing to the significant advancements in identifying genetic components of g-NDDs via next-generation sequencing technology, especially the popularization of trio-based (parental-offspring model) whole-exome sequencing (trio-WES) that enable the detection of exon-level variants, including SNVs and CNVs, genetic causes are being found more frequently than before in many g-NDDs patients (6). Nonetheless, there still have children with g-NDDs remain undiagnosed after undergoing trio-WES due to a proportion of variants located outside exons (e.g., intron, promoter or enhancer-level variants) underlying g-NDDs (7); the diagnostic yield of trio-WES for children with g-NDDs is still not satisfactory (8–10). Considering the unsatisfactory diagnostic efficacy of trio-WES in g-NDDs, it is crucial for clinicians to develop a targeted approach for the early identification of patients who can most likely be diagnosed by trio-WES, providing those patients with further assessment of related medical conditions earlier in the disease course. Moreover, owing to the exon-level sequencing feature of trio-WES, developing a simple-to-use tool for patients and their families to assist their decision-making in applying trio-WES in the diagnostic strategy at the pre-diagnosis stage is also critical, which can dramatically help facilitate individualized family medical planning.

Alignment diagrams are one of the common graphical calculators used to visualize the scoring system of a linear regression model (such as logistic and cox regression models) with an operationally intuitive manner (11). Owing to the rapid development of many R packages, such as “rms” and “rmda” packages (12), alignment diagrams can be directly generated in the R environment and represent a prevalent and major subtype within the broader category of graphical calculators, characterized by straight scales and solution via a straightedge alignment line (i.e., drawing a straight line between known values on two scales to read the unknown value on a third scale), and providing a high-level snapshot of regression accuracy. Alignment diagram models are widely applied in forecasting the risks or outcomes of many NDDs, such as ADHD (13), ASD (14), infant neurodevelopmental delays (15) and oppositional defiant disorder (16). However, to the best of our knowledge, no publications have reported the application of alignment diagrams to predict the diagnostic probability of trio-WES in g-NDDs children. Here, we conducted a double-center study with independent temporal (internal) and geographical (external) validations based on phenotype-driven methods, exploring key phenotypic variables for forecasting the diagnostic probability of trio-WES in children with g-NDDs. Phenotype-driven methods are important clinical genetic methods for identifying the key clinical phenotypic characteristics of rare monogenic disorders, allowing the identification of individuals with a high probability of carrying relevant exon-level variants detected by trio-WES (17, 18).

## Materials and methods

### Participants and patient selection criteria

**Figure 1:**
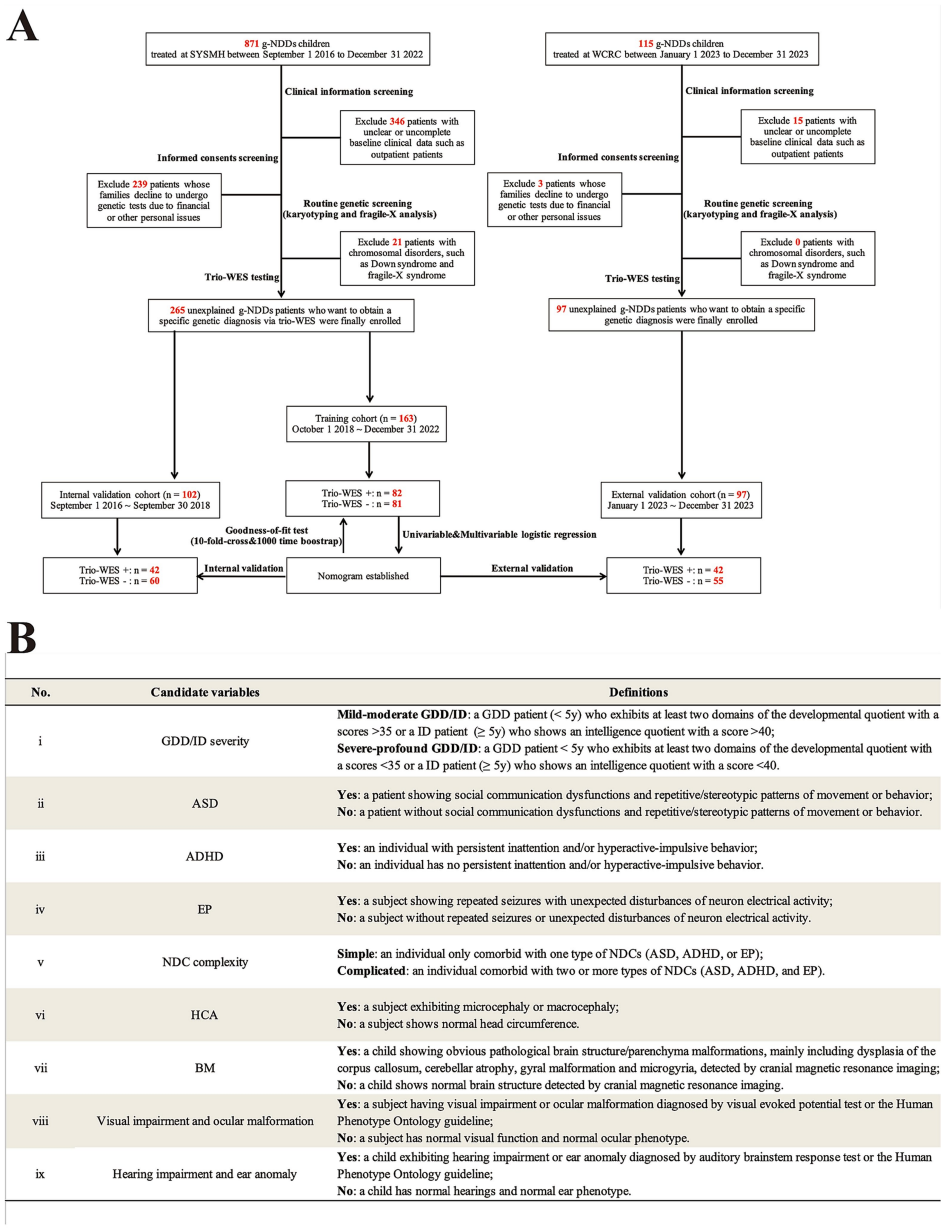
Flowchart and candidate variables description of this double-center study. **(A)** Flowchart of this study. **(B)** A description summary of candidate phenotypic variables applied in current study. g-NDDs, genetic neurodevelopmental disorders; trio-WES, trio-based whole-exome sequencing; SYSMH, Sun Yat-Sen Memorial Hospital; WCRC, Weierkang Children’s Rehabilitation Center; GDD/ID, global developmental delay/intellectual disability; ASD, autism spectrum disorder; ADHD, attention deficit hyperactivity disorder; EP, epilepsy; NDC, neurodevelopmental comorbidity; HCA, head circumference abnormality; BM, brain malformation. Note: “+/−” indicates enrolled individuals who received trio-WES and had a positive genetic diagnosis (carrying a “likely pathogenic” or “pathogenic” variant) or a negative genetic diagnosis (carrying a “benign” or “uncertain significance” variant) on the basis of the guidelines of the American College of Medical Genetics.

As shown in Figure 1A, 871 g-NDDs children were admitted to the tertiary Children’s Medical Center of Sun Yat-sen Memorial Hospital (SYSMH) from September 2016 to December 2022. After performing a series of clinical information (excluding 346 patients without clear or complete clinical data, such as outpatient subjects), informed consent (excluding 239 patients due to their families decline to receive genetic tests), and routine genetic (G-band karyotyping and fragile-X analysis) screenings (excluding 21 patients who had apparent chromosomal disorders, such as Down syndrome and fragile-X syndrome, which had no need to use high-throughput sequencing, such as trio-WES, as their diagnostic strategies), a total of 265 g-NDDs patients with non-consanguineous relationships from SYSMH who want to get a specific genetic diagnosis via trio-WES were recruited for this retrospective study. They received trio-WES testing from September 1, 2016 to December 31, 2022. Among them, 163 individuals recruited between October 1, 2018 and December 31, 2021, served as training subjects, whereas another 102 individuals with non-consanguineous relationships recruited between September 1, 2016 and September 30, 2018, served as internal validation subjects (also called “temporal validation subjects”). Moreover, 115 individuals diagnosed with g-NDDs were admitted to Weierkang Children’s Rehabilitation Center (WCRC) between January 2023 and December 2023. After a series of screenings (excluding 15 patients without clear or complete clinical data and 3 patients whose families decline to undergo genetic tests), a total of 97 non-consanguineous individuals with g-NDDs were ultimately recruited. They received trio-WES testing from January 1, 2023 to December 31, 2023, and served as the external validation subjects (also called “geographical validation subjects”).

The clinical (phenotypic) definitions of g-NDDs used in the current study included the following: (1) various severities of GDD/ID. The clinical diagnostic criteria for GDD/ID were based on the Diagnostic and Statistical Manual of Mental Disorders, 5th Edition guidelines for GDD/ID (1). The severity assessment for GDD/ID was based on the classification criteria of the International Classification of Diseases Version 11 (19). (2) Comorbid with one or more type(s) of common neurodevelopmental comorbidity (NDC), including ASD, ADHD, and EP. Clinical diagnoses of ASD and ADHD were made on the basis of the related DSM-V guidelines for ASD and ADHD (1). The clinical diagnosis of EP was made on the basis of the International League Against Epilepsy guidelines (20). (3) Patients with identified non-genetic causes, such as hypoxic–ischemic encephalopathy, bilirubin encephalopathy and intrauterine infections, were excluded.

The inclusion criteria for this study were as follows: (1) g-NDDs individuals who were admitted to SYSMH (Sep 2016 ~ Dec 2022) or WCRC (Jan 2023 ~ Dec 2023). (2) Underwent systematic and standard examinations, including assessing clinical manifestations/detailed medical history (including GDD/ID condition, history of ASD, EP and ADHD, birth condition and family history), and receiving cardinal neurodevelopment-related auxiliary examinations (Gesell Developmental Scale for Infants/Wechsler Intelligence Scale for Children, electroencephalogram, cranial magnetic resonance imaging, auditory brainstem response/visual evoked potential tests, echocardiography/abdominal ultrasonography). (3) The patients’ families agreed to undergo genetic tests, including routine genetic screening (G-band karyotyping and fragile-X analysis) and trio-WES, and want to obtain a specific genetic diagnosis through trio-WES.

The exclusion criteria for this study were as follows: (1) g-NDDs patients whose medical records were incomplete or unclear or whose clinical data were missing. (2) Positive routine genetic test results indicated that there were apparent chromosomal disorders, such as Down syndrome or fragile-X syndrome, in those individuals, which had no need to use trio-WES as a diagnostic strategy in those patients. (3) Those who underwent trio-WES analysis but did not permit the use of their trio-WES results for publication.

### Ethical compliance

The design and launch of this retrospective study were approved by the Ethical Committee of the Sun Yat-sen Memorial Hospital, Sun Yat-sen University (Approval Number: SYSKY-2025-244-01). Written informed consents for genetic investigation and participation in this study were obtained from the parents or guardians of all 362 enrolled individuals.

### Methods for variant capture of trio-WES

The principle of variant capture process and quality control system of trio-WES have been described in previous studies (21–23) and the methods in this study can be briefly presented as follows: DNAs were extracted from the whole blood of the proband and their parents using a commercialized genomic extraction kit (Qiagen, Shanghai, China). Illumina TruSeq Exome Kit (Illumina, San Diego, CA, United States) was used for the DNA library construction and the generation of ~10GB exome sequencing data/individual. GeneRanger (Xunyin Biotech, Shanghai, China) was employed for the exome sequencing data analysis. Then, a series of the genome analysis tools were employed for the read alignment, indel region realignment, base quality recalibration, variant capture, and calling/transformation on the basis of the Genome Aggregation Database (gnomAD). Variant capture control system was set to a coverage depth >10 with a minor allele frequency <0.05%.

### Criteria for identifying exon-level variant pathogenicity assessment and subject grouping

The pathogenicity of the detected exon-level SNVs via trio-WES was scored on the basis of the 2015 American College of Medical Genetics guidelines for SNVs classification (24), and those candidate SNVs were accordingly divided into “pathogenic/likely pathogenic SNVs” and “benign/uncertainly significant SNVs.” As described in previous research (5), gnomAD and the in-house SNV population frequency databases were employed for the assessment of SNVs allele frequency. Functional analysis of the pathogenicity of those variants was performed via bioinformatic tools. Specifically, *in silico* prediction of identified missense/frameshift/nonsense/deletion variants’ pathogenicity was conducted using local versions of Mutation Taster, PROVEAN, Polyphen-2, REVEL and SIFT. Moreover, *in silico* pathogenic prediction of detected splice variants was conducted using local versions of CADD and Human Splice Finder. Human Genomic Mutation Database and PubMed were employed to determine whether identified variants had been recorded previously. GeneReviews and OMIM databases were employed to obtain the genotype–phenotype profiles linked to identified SNVs. The pathogenicity of exon-level CNVs identified via trio-WES was rated on the basis of the 2019 American College of Medical Genetics guidelines for the interpretation of postnatal CNVs (25), and those candidate CNVs were accordingly classified as “pathogenic/likely pathogenic CNVs” and “benign/uncertainly significant CNVs.” All candidate SNVs/CNVs in this study were manually interpreted and assessed by two or more experienced clinical geneticists, following the American College of Medical Genetics guidelines mentioned above. ClinVar database^1^ was employed to refer the report status (novel or previously reported) of all identified SNVs and CNVs.

On the basis of the outcome indicator from the trio-WES examination records of the hospital digital system, the enrolled individuals were then grouped into those with positive trio-WES-tested genetic diagnoses (i.e., those with pathogenic/likely pathogenic exon-level SNVs or CNVs in their trio-WES analysis reports) and those with negative trio-WES-tested genetic diagnoses (i.e., those with benign/uncertainly significant exon-level SNVs or CNVs in their trio-WES analysis reports and maybe the out-exon-level variants can explain their g-NDDs conditions).

### Candidate variable collection and interpretation of collected variables

The demographic characteristics and phenotypic variables of all enrolled subjects were collected from medical records of the hospital digital system. This included (1) demographic characteristics, such as sex, onset age, and admission date. (2) candidate phenotypic variables: (i) GDD/ID severity; (ii) ASD; (iii) ADHD; (iv) EP; (v) NDC complexity; (vi) Head circumference abnormality (HCA); (vii) Brain structure malformation (BM); (viii) Visual impairment and ocular malformation; (ix) Hearing impairment and ear anomaly. A summary of those phenotypic variables and their detailed definitions were demonstrated in Figure 1B.

### Model visualization and reliability and performance evaluation

Independent phenotypic variables were determined via univariate and multivariate logistic regression analysis using the data from the training cohort. Specifically, during the univariate and multivariate regression analyses of the training cohort, variables with clinical significance and significant differences (*p* < 0.05) were identified between groups with positive and negative trio-WES-based genetic diagnoses, and used for logistic linear model construction.

Firstly, we used Spearman’s correlation analysis to determine whether there were confirmed bi-interdependencies among the identified phenotypic variables. According to the classification of statistical power for correlation analysis established by Cohen (26), when the absolute value of Spearman’s correlation coefficient (| *r* |) < 0.5 for all pairwise comparisons among those variables, there were no confirmed bi-interdependencies existed among them. Then, we used collinearity analysis to further determine whether there were significant multiple interdependencies among those phenotypic factors. Specifically, the tolerance and variance inflation factor (VIF) were applied to assess the multiple interdependence among identified variables. When the value of tolerance was >0.2 and the VIF was <2 for each identified variable, there were no multiple interdependencies existed among those variables, and could be considered independent phenotypic variables for linear model construction (27). Then, we used the 10-fold-cross resampling with the 1,000-time bootstrap repeated sampling methods to evaluate the goodness-of-fit and reliability degrees of the linear regression model in training set. Specifically, the 10-fold-cross resampling approach is an established and robust resampling technique employed to assess the consistence performance and the reliability of constructed model, which involves partitioning the entire dataset into 10 mutually exclusive and approximately equal-sized folds. During iterative training, 9 folds are aggregated as the training subset; the remaining single fold serves as the validation subset. This process is repeated 10 times with each fold served exactly once as the validation subset. Meanwhile, the bootstrap resampling method is another classical approach that is drawn from the original training dataset with replacement and is repeated 1,000 times. The established performance metric, concordance index (C-index) of the two methods, is calculated to examine the reliability of constructed model; when the two methods’ C-index values were >0.7, indicating the regression model can be regarded as a good reliability linear model for explaining variance of the set outcomes (28). Finally, we generated an alignment diagram using R packages to visualize the scoring system of our constructed logistic regression model.

For model performance evaluation, the discriminatory power was first evaluated in the training set via the area under the curve (AUC) values of the receiver operating characteristic (ROC) curves. Then, the calibration curves with the Hosmer–Lemeshow (H-L) test were used to evaluate the goodness of fit between the predicted calculation and the observed data. Specifically, when the *p*-value of H-L test <0.05, the dotted line (representing model-predicted data) in calibration curve significantly differed from the solid line (representing actual observed data) in calibration curve, and the model did not fit well. Otherwise, the model fit well (*p*-value of H-L test >0.05). Moreover, the benefit of the clinical application of the model was also assessed via decision curve analysis (DCA) and a clinical impact curve (CIC).

### Model performance validation

According to the maximal Youden index value (corresponding to the most optimal values of sensitivity and specificity of model) calculated in the training set, we further set the corresponding individual’s total score (cut-off score) for the training and internal/external validation cohorts, then based on this cut-off score, we further divided subjects in training and internal/external validation sets into a “model-predicted high probability diagnostic group” and a “model-predicted low probability diagnostic group.” The AUC values of the ROC curves, calibration curves with the H-L test and DCA/CIC were subsequently applied to verify the discriminative performance of model in internal and external validation sets. Finally, we calculated the model sensitivity, specificity, accuracy, precision and F1 score for the training and external validation sets and visualized those results (confusion matrix) via Sankey plots.

### Statistical analysis

In this study, Microsoft Excel software was used to record individual data, and all the statistical analyses were conducted in the R environment (version 4.4.2, https://www.r-project.org/). As in previous studies in the R environment (12, 27, 29), the main R packages used for statistical analysis and figure plotting in the present research included “ggplot2,” “foreign,” “rms,” “rmda,” “caret,” “tidyverse” and “ggDCA.” The results with a *p*-value <0.05 were considered statistically significant throughout the study, if not specially noted.

## Results

### Patient characteristics

In total, 163, 102, and 97 pediatric and adolescent subjects with unexplained g-NDDs were enrolled in the training, internal validation, and external validation cohorts, respectively. A comparison of the baseline demographics and phenotypic characteristics of the subjects in the training and validation cohorts is shown in Table 1, and the detailed genotypic and phenotypic data of those individuals in the training and validation cohorts can be found in Supplementary Files 1–6, respectively.

**Table 1:** Comparison of baseline demographics or phenotypic characteristics between training cohort and internal/external validation cohorts of trio-WES tested children with g-NDDs.

Demographics or characteristic	Indicators	Training cohort	Internal validation cohort	*t/χ*^2^ value	*p*-value*	External validation cohort	*t/χ*^2^ value	*p*-value**
Demographic parameters								
Case number (n)		163	102			97		
Sex [*n* (%)]	Female	57 (35.0%)	26 (25.5%)	2.199	0.138	28 (28.9%)	0.771	0.380
	Male	106 (65.0%)	76 (74.5%)			69 (71.1%)		
Admission Periods (MM/YY ~ MM/YY)		Oct/2018 ~ Dec/2022	Sep/2016 ~ Sep/2017			Jan/2023 ~ Dec/2023		
Onset age [Mean ± SEM/(y)]		4.396 ± 0.261	4.610 ± 0.428	0.428	0.669	4.945 ± 0.300	1.380	0.169
Patient sources		SYSMH	SYSMH			WCRC		
Phenotypic variates								
GDD/ID severity [*n* (%)]	Mild–moderate	93 (57.1%)	63 (61.8%)	0.397	0.529	63 (64.9%)	1.267	0.260
	severe-profound	70 (42.9%)	39 (38.2%)			34 (35.1%)		
NDC complexity [*n* (%)]	Simple	129 (79.1%)	81 (79.4%)	0.000	1.000	69 (71.1%)	1.729	0.189
	Complicated	34 (20.9%)	21 (20.6%)			28 (28.9%)		
ASD [*n* (%)]	Yes	99 (60.7%)	69 (67.6%)	1.011	0.315	58 (59.8%)	0.000	0.985
	No	64 (39.3%)	33 (32.4%)			39 (40.2%)		
ADHD [*n* (%)]	Yes	41 (25.2%)	24 (23.5%)	0.023	0.879	33 (34.0%)	1.933	0.164
	No	122 (74.8%)	78 (76.5%)			64 (66.0%)		
EP [*n* (%)]	Yes	62 (38.0%)	42 (41.2%)	0.259	0.611	35 (36.1%)	0.099	0.753
	No	101 (62.0%)	60 (58.8%)			62 (63.9%)		
HCA [*n* (%)]	Yes	44 (27.0%)	33 (32.4%)	0.633	0.426	27 (27.8%)	0.000	0.997
	No	119 (73.0%)	69 (67.6%)			70 (72.2%)		
BM [*n* (%)]	Yes	23 (14.1%)	24 (23.5%)	3.197	0.074	8 (8.2%)	1.471	0.225
	No	140 (85.9%)	78 (76.5%)			89 (91.8%)		
Visual impairment/ocular malformation [*n* (%)]	Yes	8 (4.9%)	2 (2.0%)	0.799	0.371	10 (10.3%)	1.979	0.160
	No	155 (95.1%)	100 (98.0%)			87 (89.7%)		
Hearing impairment/ear anomaly [*n* (%)]	Yes	22 (13.5%)	6 (5.9%)	3.086	0.079	9 (9.3%)	0.668	0.414
	No	141 (86.5%)	96 (94.1%)			88 (90.7%)		
Outcomes								
Trio-WES-based diagnostic status [*n* (%)]	Positive	82 (50.3%)	42 (41.2%)	1.750	0.186	42 (43.3%)	0.933	0.334
	Negative	81 (49.7%)	60 (58.8%)			55 (56.7%)		

*, training cohort vs. internal validation cohort; **, training cohort vs. external validation cohort; g-NDDs, genetic neurodevelopmental disorders; trio-WES, trio-based whole exome sequencing; SYSMH, Sun Yat-sen Memorial Hospital; WCRC, Weierkang Children’s Rehabilitation Center; GDD/ID, global developmental delay/intellectual disability; NDC, neurodevelopmental comorbidity; ASD, autism spectrum disorder; ADHD, attention deficit hyperactivity disorder; EP, epilepsy; HCA, head circumference abnormality; BM, brain malformation.

Among the 163 enrolled subjects in the training cohort, 93 (57.1%) had mild–moderate GDD/ID, and the remainder (70/163, 42.9%) had severe-profound GDD/ID. Most children (129/163, 79.1%) presented as simple g-NDDs without comorbid ASD, ADHD or EP. Meanwhile, parts of cases had ASD (99/163, 60.7%), ADHD (41/163, 25.2%), EP (62/163, 38.0%), HCA (44/163, 27.0%), BM (23/163, 14.1%), visual impairment/ocular malformation (8/163, 4.9%) and hearing impairment/ear anomaly (22/163, 13.%), respectively. 82 (50.3%) had a genetic diagnosis via trio-WES. Of the 82 trio-WES diagnosed children in the training cohort, 62 (75.6%) cases were diagnosed with SNV-mediated syndromes (a total of 72 detected SNVs with 35 being novel variant and 37 being previously reported) and 20 (24.4%) with CNV-mediated syndromes (all these 20 identified CNVs were novel variants).

Among the 102 and 97 enrolled cases in the internal and external validation cohorts, 42 (41.2%) individuals included in the internal validation cohort obtained a genetic diagnosis via trio-WES. Of the 42 trio-WES diagnosed children, 36 (85.7%) cases were diagnosed with SNV-mediated syndromes (a total of 40 detected SNVs with 14 being novel variant and 26 being previously reported) and 6 (14.3%) cases were diagnosed with CNV-mediated syndromes (all these 6 identified CNVs were novel variants). While, in external validation cohort, 42 (43.3%) enrolled subjects received a genetic diagnosis via trio-WES. Of the 42 trio-WES diagnosed children, 32 (76.2%) cases were diagnosed with SNV-mediated syndromes (a total of 34 detected SNVs with 16 being novel variant and 18 being previously reported) and 10 cases (23.8%) diagnosed with CNV-mediated syndromes (all these 10 identified CNVs were novel variants).

### Independent predictive indicator selection and regression model construction

First, univariate logistic analysis for all included phenotypic variables presented in Table 1 was performed in the training set to determine the potential trio-WES diagnosis-related phenotypic markers. As demonstrated in Table 2, the results of univariate logistic analysis revealed that four candidate phenotypic markers (GDD/ID severity, NDC complexity, ASD, and HCA) had potential predictive value. Among them, severe-profound GDD/ID [OR (95% CI): 6.880 (3.414–13.865), *p* < 0.001], multiple NDCs [OR (95% CI): 4.237 (1.783–10.067), *p* < 0.01], ASD [OR (95% CI): 4.673 (2.360–9.253), *p* < 0.001], and HCA [OR (95% CI): 4.286 (1.977–9.292), *p* < 0.001] were associated with high possibilities of having genetic diagnosis via trio-WES in g-NDDs subjects.

**Table 2:** Univariate and multivariate logistic regression for predicting diagnostic efficacy of using trio-WES in g-NDDs individuals in training set.

Univariate logistic analysis			Multivariate logistic analysis		
Candidate indicators	OR (95%CI)	*p*-value	Candidate indicators	OR (95%CI)	*p*-value
GDD/ID severity	6.880 (3.414–13.865)	<0.001***	GDD/ID severity	4.865 (2.213–10.694)	<0.001***
NDC complexity	4.237 (1.783–10.067)	0.001**	NDC complexity	3.731 (1.399–9.950)	0.009**
ASD	4.673 (2.360–9.253)	<0.001***	ASD	3.256 (1.479–7.168)	0.003**
ADHD	1.196 (0.589–2.431)	0.620			
EP	0.580 (0.306–1.100)	0.095			
HCA	4.286 (1.977–9.292)	<0.001***	HCA	2.788 (1.148–6.774)	0.024*
BM	2.043 (0.814–5.126)	0.128			
Visual impairment/ocular malformation	3.118 (0.610–15.932)	0.172			
Hearing impairment/Ear anomaly	1.217 (0.494–2.999)	0.669			

Trio-WES, trio-based whole exome sequencing; g-NDDs, genetic neurodevelopmental disorders; GDD/ID, global developmental delay/intellectual disability; NDC, neurodevelopmental comorbidity; ASD, autism spectrum disorder; ADHD, attention deficit hyperactivity disorder; EP, epilepsy; HCA, head circumference abnormality; BM, brain malformation; OR (95%CI), odds ratio (95% confidence interval). *p < 0.05, **p < 0.01, ***p < 0.001.

**Figure 2:**
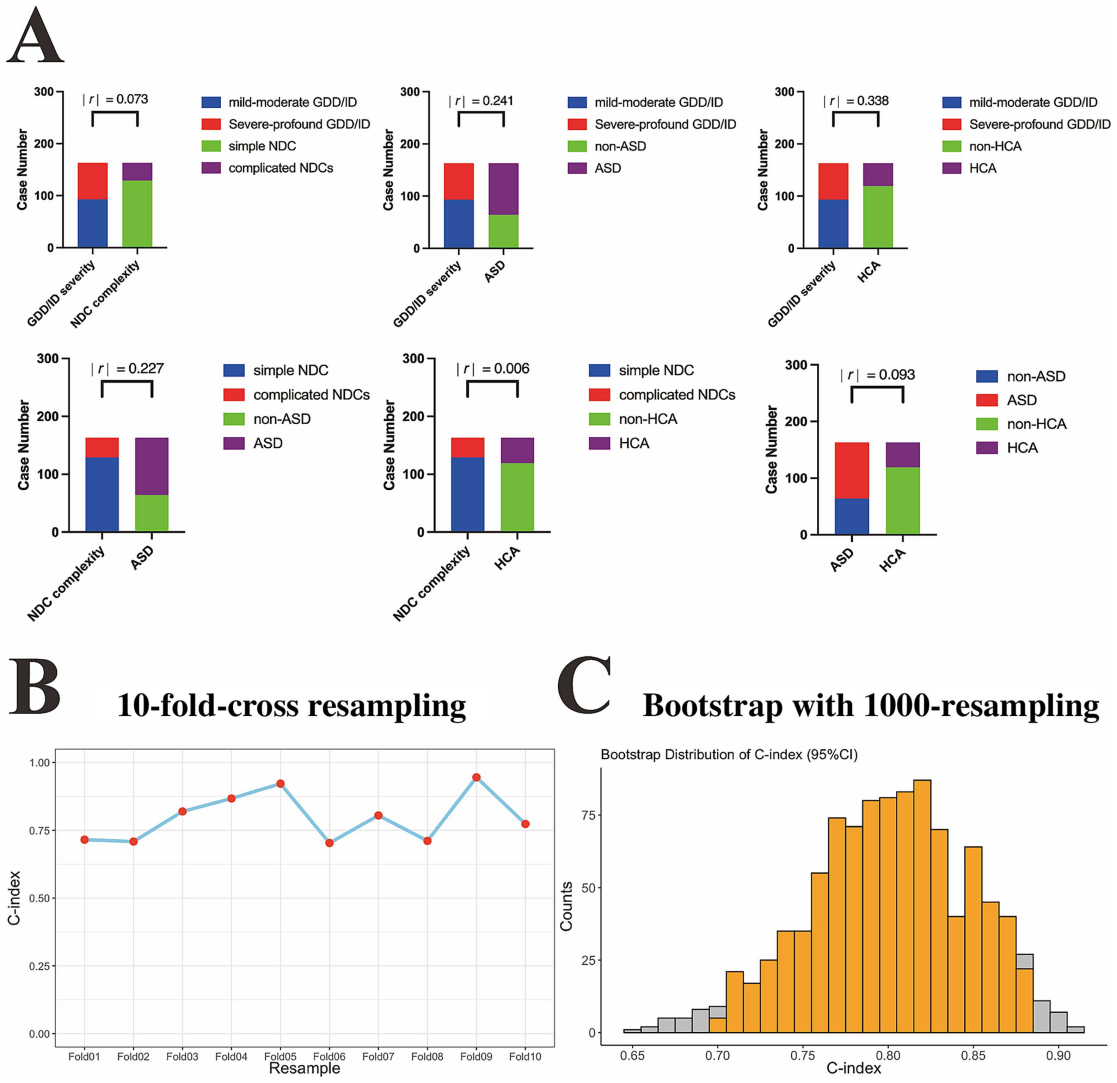
Spearman’s correlation analysis and Goodness-of-fit tests for evaluation of the reliability of the constructed logistic regression model. **(A)** Bar charts showing the pairwise comparisons of |*r*| values among those model-constructed variables across training cohort. **(B)** Point-fold line chart with 10-fold-cross resampling approach showing the model had good stability with excellent consistence in training set (C-index, 0.797 with 95% CI, 0.732–0.862). **(C)** Histogram with 1,000-time resampling bootstrap method revealing the model did not overfit and showed good reliability in training set (C-index, 0.800 with 95% CI, 0.707–0.893). |*r*|, Spearman’s correlation coefficient; GDD/ID, global developmental delay/intellectual disability; ASD, autism spectrum disorder; NDC, neurodevelopmental comorbidity; HCA, head circumference abnormality.

On the basis of the univariate regression analysis, the phenotypic factors with *p* < 0.05, including GDD/ID severity, NDC complexity, ASD, and HCA (Figure 1B), were candidate indicators for multivariate linear regression model construction. The four variables were firstly analyzed via Spearman’s correlation analysis. As shown in Figure 2A, the values of |*r*| for pairwise comparisons among those four variables were all <0.5, indicating that no confirmed bi-interdependencies existed among them. Then, the four indicators were subsequently analyzed via collinearity analysis. As demonstrated in Table 3, the tolerances of each phenotypic factor were all >0.2, and the values of the VIFs of each indicator were all <2, indicating that no multiple interdependencies existed among those four phenotypic variables.

**Table 3:** The collinearity diagnostic analysis of variables for predicting efficacy of using trio-WES in g-NDDs individuals in training set.

Candidate variables	Tolerance	VIF
GDD/ID severity	0.841	1.189
NDC complexity	0.947	1.056
ASD	0.898	1.114
HCA	0.884	1.131

Trio-WES, trio-based whole exome sequencing; g-NDDs, genetic neurodevelopmental disorders; GDD/ID, global developmental delay/intellectual disability; NDC, neurodevelopmental comorbidity; ASD, autism spectrum disorder; HCA, head circumference abnormality; VIF, variance inflation factor.

Those four phenotypic factors without interdependencies were incorporated into the multivariate logistic regression. As indicated in Table 2, our multivariate analysis results further revealed that GDD/ID severity [OR (95% CI): 4.865 (2.213–10.694), *p* < 0.001], NDC complexity [OR (95% CI): 3.731 (1.399–9.950), *p* = 0.009], ASD [OR (95% CI): 3.256 (1.479–7.168), *p* = 0.003] and HCA [OR (95% CI): 2.788 (1.148–6.774), *p* = 0.024] were independently associated with the diagnostic efficacy of trio-WES among g-NDDs subjects. Then, we used the 10-fold-cross validation and the bootstrap repeated sampling approach for evaluation the reliability of constructed model. As shown in Figure 2B, after conducting 10-fold-cross validation, the C-index value was 0.797 with a 95% CI of 0.732–0.862. In the meantime, after performing bootstrap method with 1,000-time resampling, the C-index also show robust value with 0.800 (95% CI: 0.707–0.893) (Figure 2C). All those results (both two approaches’ C-indices were >0.7) indicated that the constructed regression model owned good goodness-of-fit and can be regarded as a reliability linear model for explaining variance of the set outcome (diagnosed by trio-WES) for g-NDDs patients.

According to the multivariate logistic regression *β* value and the intercept term, a novel regression model for predicting the diagnostic efficacy of using trio-WES in pediatric patients with g-NDDs was constructed, and the corresponding formula for predicting the probability (*P*) of a g-NDDs subject being diagnosed by trio-WES was as follows: Logit (P) = 1.582 (β_1_) × GDD/ID severity (severe-profound: 1; mild–moderate: 0) + 1.317 (β_2_) × NDC complexity (complicated: 1; simple: 0) + 1.181 (β_3_) × ASD (yes: 1; no: 0) + 1.025 (β_4_) × HCA (yes: 1; no: 0) – 1.914 (intercept term). The most prevalent scoring methodology for included variables is utilization of the coefficients (β) derived from regression model. The calculated score assigned to each variable is proportional to its β value, often mapped to a 0 to 100-point scale via linear transformation (30). Specifically, we set 100-point to a variable with the max β value (β_max_), and based on that, we can calculate other included variables’ calculated score via formula “calculated score_x_ = 100 × β_x_÷β_max_.” Detailed coefficients of this regression model and the corresponding calculated score for each included variable were demonstrated in Table 4.

**Table 4:** Coefficients of binary logistic regression for predicting diagnostic efficacy via trio-WES in children with g-NDDs in training set.

Phenotypic variables	*B*	S. E.	Wald	*p*-value	OR	95% CI for OR	Calculated score
GDD/ID severity	1.582 (β1)	0.402	15.500	<0.001	4.865	2.213–10.694	100*
NDC complexity	1.317 (β2)	0.500	6.921	0.009	3.731	1.399–9.950	83 (100 × β2÷*β*1)
ASD	1.181 (β3)	0.403	8.601	0.003	3.256	1.479–7.168	75 (100 × β3÷β1)
HCA	1.025 (β4)	0.453	5.124	0.024	2.788	1.148–6.774	65 (100 × β4÷β1)

Trio-WES, trio-based whole exome sequencing; g-NDDs, genetic neurodevelopmental disorders; GDD/ID, global developmental delay/intellectual disability; NDC, neurodevelopmental comorbidity; ASD, autism spectrum disorder; HCA, head circumference abnormality; B, *β* value; S. E., standard error; OR, odds ratio; 95% CI, 95% confidence interval. *The variable showing the max *β* value and being set 100-point as reference-point for other included variables.

### Model visualization via alignment diagram and the assessment of the related visualized scoring system

**Figure 3:**
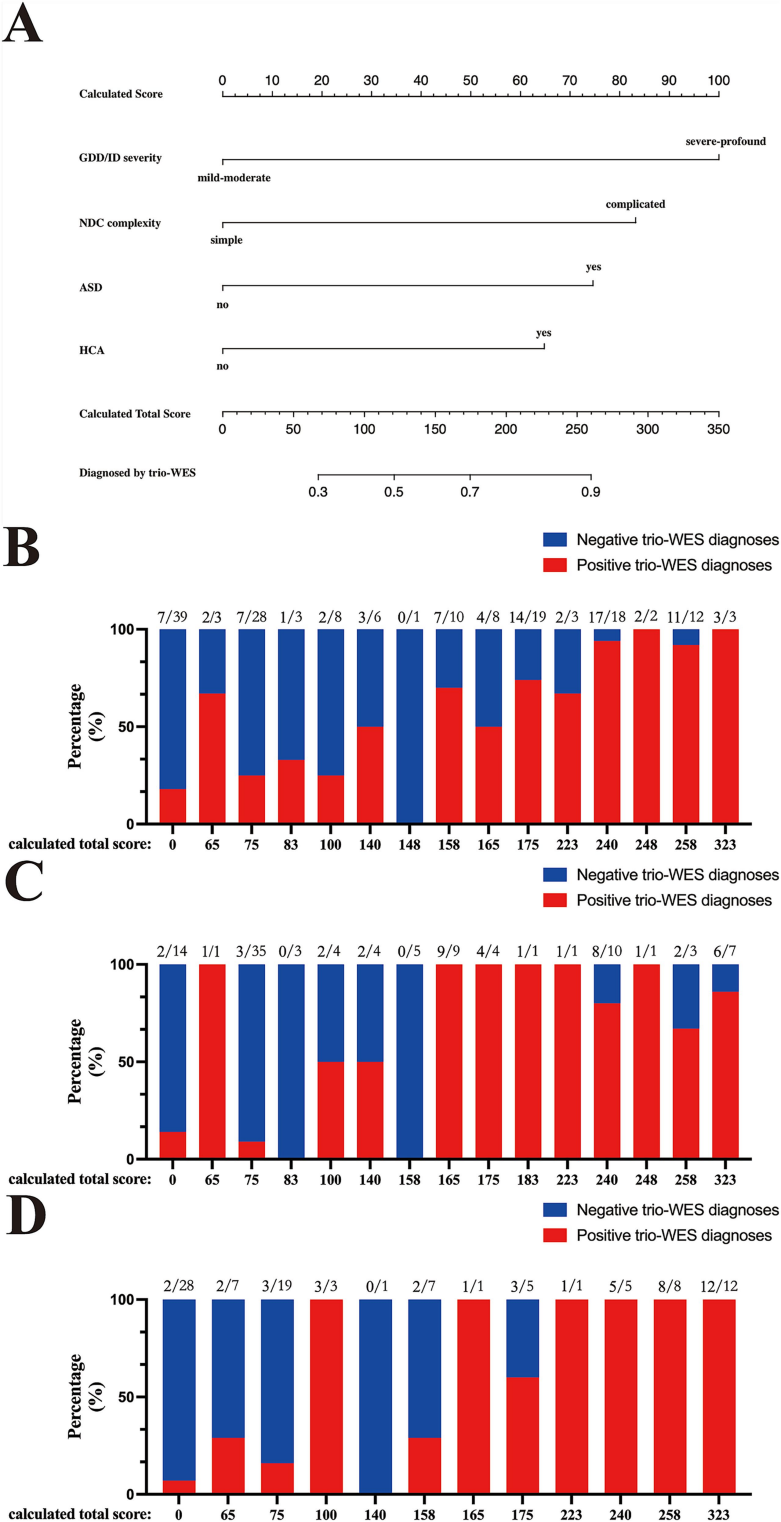
Predictive model visualization by the alignment diagram and the assessment of this visualized scoring system across the training and validation cohorts. **(A)** The alignment diagram includes two parts: the top part (starts with “Calculated Score” and goes down to the last phenotypic factor, “HCA”) is used to calculate the scores of each included phenotypic biomarker, and the bottom part (starts with “Calculated Total Scores” and goes down to “Diagnosis by trio-WES”) is designed to determine the probability of having a genetic diagnosis via trio-WES. Bar charts revealing the percentage distributions of cases with positive trio-WES diagnoses at each score across the training **(B)**, internal **(C)** and external **(D)** validation cohorts. GDD/ID, global developmental delay/intellectual disability; NDC, neurodevelopmental comorbidity; ASD, autism spectrum disorder; HCA, head circumference abnormality; trio-WES, trio-based whole-exome sequencing; g-NDDs, genetic neurodevelopmental disorders.

As shown in Figure 3A, by using R packages “rms” and “rmda,” we generated an alignment diagram to visualize the constructed logistic regression model and its related scoring system (Table 4). Specifically, the alignment diagram provided an estimate of the probability of being diagnosed by trio-WES via the scored contributions of the four included binary phenotypic indicators and could be used for an g-NDDs child at the time of initial admission. According to the scoring system visualized by the alignment diagram and distinct phenotypic manifestations of all enrolled 362 g-NDDs subjects in training and validation cohorts, we could assign corresponding scores to each individual, thereby deriving an aggregate score for each of them. Based on their aggregate scores, we could further classify those subjects into different subgroups at each aggregate score. As revealed in Figures 3B–D, bar charts showing the percentages of cases with positive trio-WES diagnoses in different subgroups at each aggregate score across the training, internal and external validation cohorts, and we found that a higher aggregate score was consistently and strongly associated with a significantly increased rate of positive trio-WES diagnoses across all these three cohorts, further demonstrating the robust predictive power of this scoring system for identifying g-NDDs cases likely to yield a positive genetic diagnosis via trio-WES.

### Evaluation of the predictive model in the training cohort

**Figure 4:**
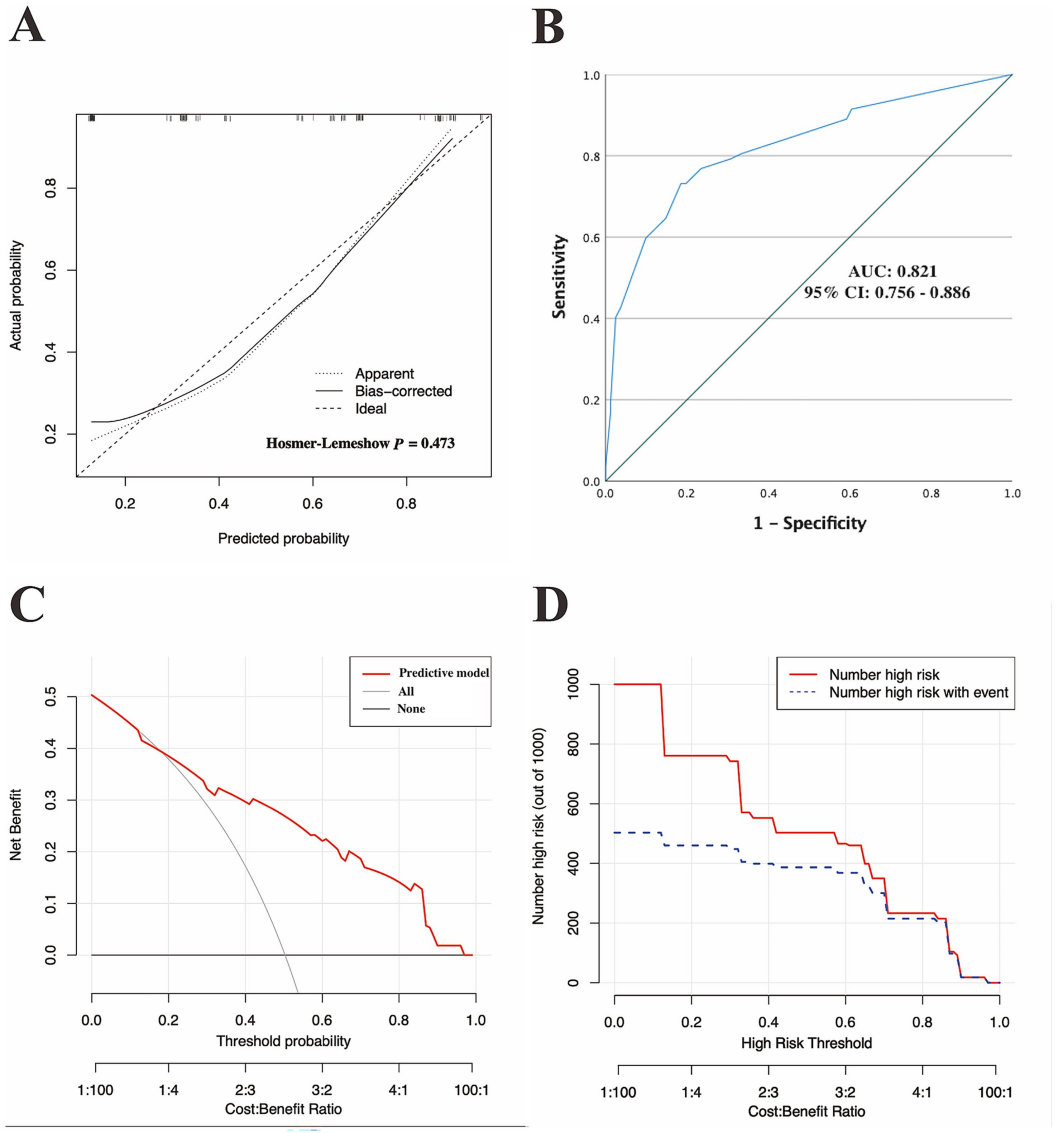
Discriminatory performance of the model in the training set. **(A)** Calibration plots, **(B)** ROC curves for assessing the predictive accuracy of the model in the training cohort, and **(C)** DCA and **(D)** CIC for examining the predicted clinical utility or impact of the model in the training set. AUC, area under the ROC curves; 95% CI, 95% confidence interval; ROC, receiver operating characteristic; DCA, decision curve analysis; CIC, clinical impact curve.

In the training set, the calibration curves with the H-L test were first used to assess the fitness of the predictive model. As revealed in Figure 4A, the calibration plots of the model showed a good fit between the actual and model-predicted diagnostic rates, and in the H-L test, *χ*^2^ = 5.571 with a *p*-value = 0.473, further indicating satisfactory consistency between the model predictions and the actual observed values.

**Figure 5:**
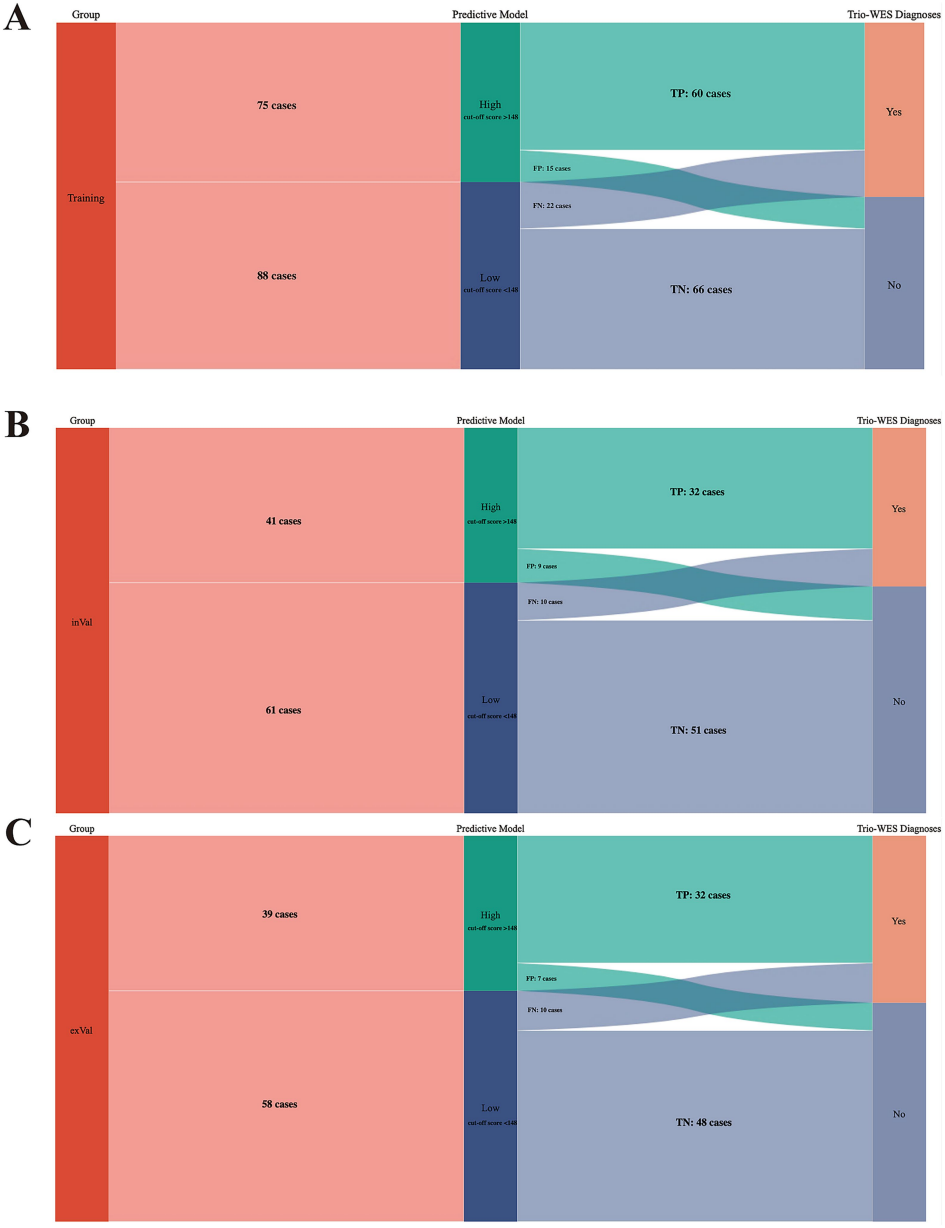
Sankey plots revealing the discriminatory performance of the predictive model across training and validation cohorts. **(A)** Training cohort, **(B)** internal validation cohort and **(C)** external validation cohort. Training, training cohort; inVal, internal validation cohort; exVal, external validation cohort. FP, false positive; TP, true positive; TN, true negative; FN, false negative. Note: the maximal Youden index (0.547) based on the training cohort was chosen to set the optimal cut-off score (148), a critical value that clustered those three cohorts (Training, inVal, and exVal cohorts) into groups with high probability and low probability of having a genetic diagnosis via trio-WES.

ROC curves were subsequently used to assess the discriminative performance of the predictive model. As shown in Figure 4B, the results demonstrated that the AUC = 0.821 (95% CI: 0.756–0.886), suggesting that the model had good predictive value in the training set. According to the ROC curves of the training cohort, the maximal Youden index was 0.547 and was selected to set the optimal cut-off score (148), which generated a confusion matrix with values of sensitivity, specificity, false negative rate (FNR), false positive rate (FPR), accuracy, precision, and F1 score of 73.20, 81.50, 26.80, 18.50, 77.30, 80.00%, and 0.76, respectively, in the training set (Figure 5A and Table 5).

**Table 5:** Predictive performance of the constructed model in training and validation sets.

Predictive values	Training set	Internal validation set	External validation set
Sensitivity (%)	73.20%	76.20%	76.20%
Specificity (%)	81.50%	85.00%	87.30%
FNR (%)	26.80%	23.80%	23.80%
FPR (%)	18.50%	15.00%	12.70%
Accuracy (%)	77.30%	81.40%	82.50%
Precision (%)	80.00%	78.00%	82.10%
F1 score	0.76	0.77	0.79

FNR, false negative rate; FPR, false positive rate.

Moreover, we also applied DCA and the CIC to evaluate the clinical usefulness of the predictive model in the training set. As demonstrated in Figures 4C,D, individuals with g-NDDs could obtain greater net benefits from our model than from the hypothetical treat-none or treat-all scenarios, suggesting that the use of this model to predict the diagnostic efficacy of trio-WES for g-NDDs children may bring clinicians more benefits.

### Internal (temporal) and external (geographical) validations of the model performance

**Figure 6:**
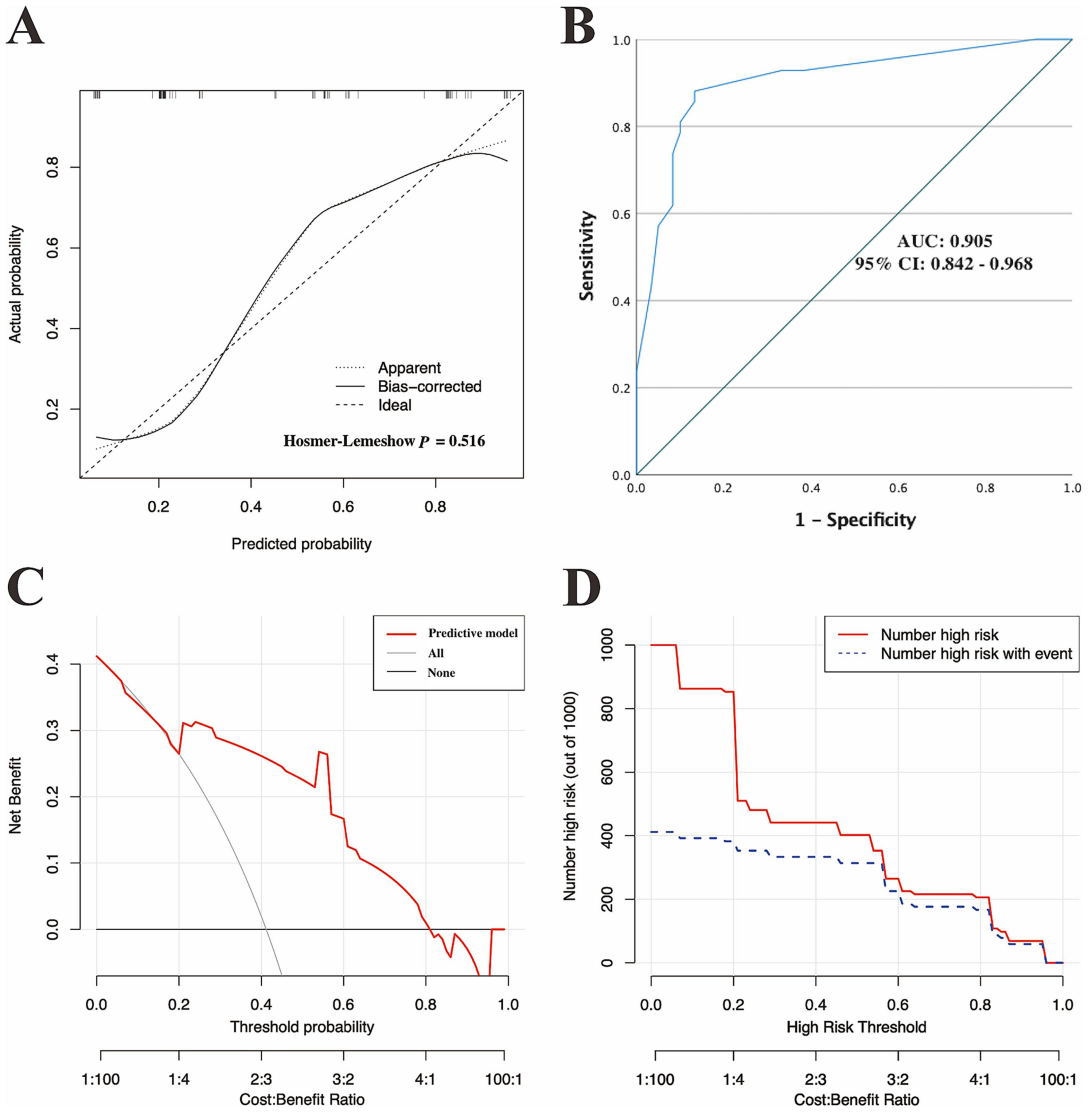
Internal verification of the discriminatory performance of the model in the internal validation set. **(A)** Calibration plots and **(B)** ROC curves verifying the estimation accuracy of genetic diagnosis via trio-WES on the basis of the predictive model. **(C)** DCA and **(D)** CIC validating the clinical value of the model in the internal validation set. AUC, area under the ROC curves; 95% CI, 95% confidence interval; ROC, receiver operating characteristic; DCA, decision curve analysis; CIC, clinical impact curve.

As shown in Figure 6A, the calibration curves with the H-L test revealed excellent agreement between the model predictions and the actual observed results (*χ*^2^ = 5.221 with *p*-value = 0.516) in the internal set. Moreover, the ROC plots also revealed that the model had a powerful discriminative ability in the internal set (Figure 6B, AUC: 0.905, 95% CI: 0.842–0.968). As reflected by the results of the DCA and CIC in the internal set, applying our model to predict the diagnostic efficacy of trio-WES for g-NDDs patients could result in greater net benefits (Figures 6C,D). As reflected by the confusion matrix results of the Sankey plot in the internal set, the sensitivity, specificity, FNR, FPR, accuracy, precision and F1 score of the model in the internal set were 76.20, 85.00, 23.80, 15.00, 81.40, 78.00%, and 0.77, respectively (Figure 5B and Table 5).

**Figure 7:**
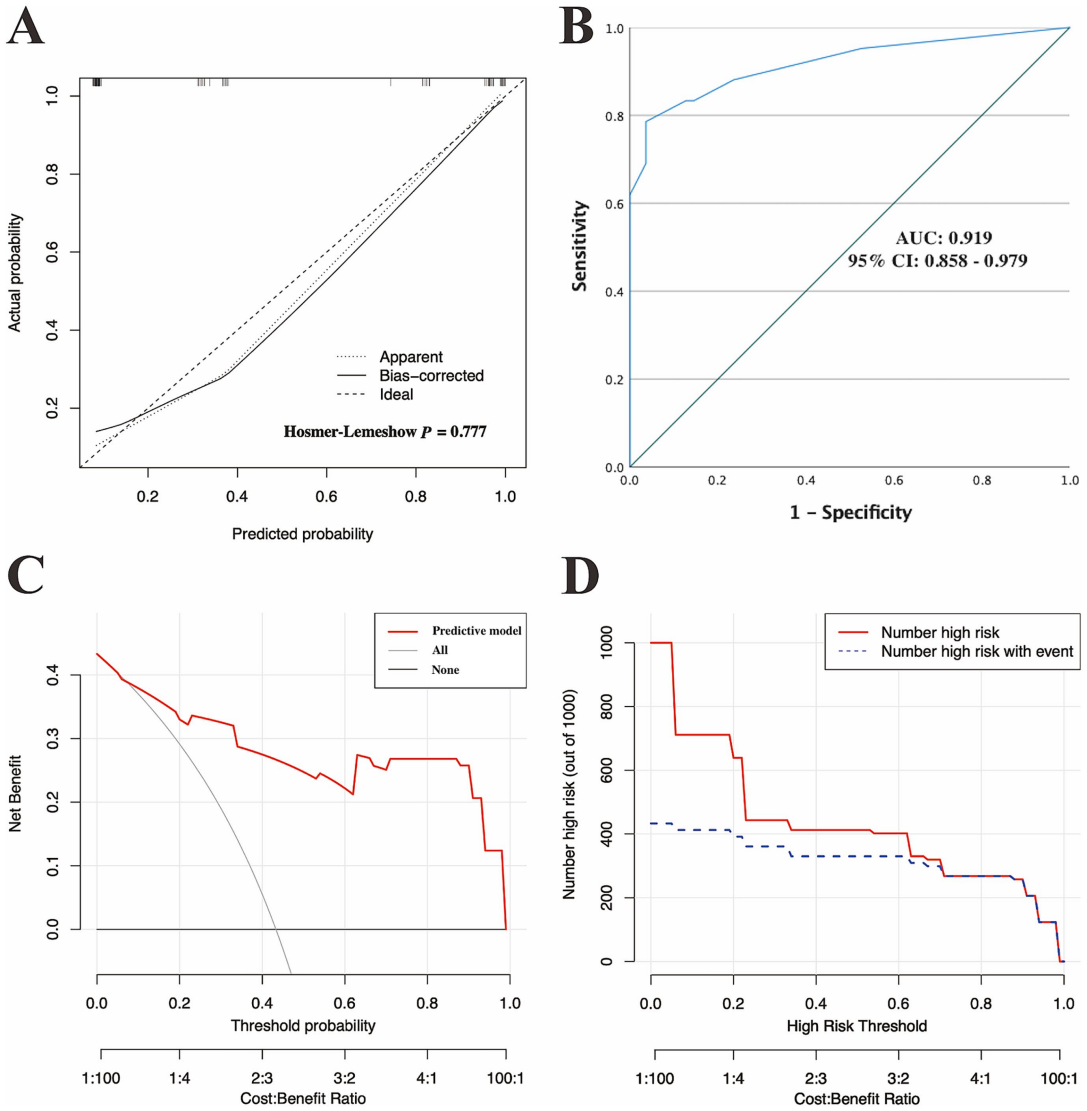
External verification of the discriminatory performance of the model in the external validation set. **(A)** Calibration plots and **(B)** ROC curves verifying the estimation accuracy of genetic diagnosis via trio-WES on the basis of the predictive model. **(C)** DCA and **(D)** CIC validating the clinical value of the model in the internal validation set. AUC, area under the ROC curves; 95% CI, 95% confidence interval; ROC, receiver operating characteristic; DCA, decision curve analysis; CIC, clinical impact curve.

Similarly, the calibration curves with the H-L test (*χ*^2^ = 2.494 with *p* value = 0.777), ROC curves (AUC: 0.919, 95% CI: 0.858–0.979) and DCA/CIC were all applied in the external set (Figures 7A–D). A confusion matrix results of the Sankey plot in the external set showing the sensitivity, specificity, FNR, FPR, accuracy, precision and F1 score of the model in the external set were 76.20, 87.30, 23.80, 12.70, 82.50, 82.10%, and 0.79, respectively (Figure 5C and Table 5).

## Discussion

With the rapid development of next-generation sequencing technology, genetic causes are being detected more frequently than before in many individuals with unexplained syndrome involving multiple organ malformations (5). The application of next-generation sequencing has completely changed the landscape of clinical genetics; compared with conventional tests (such as family segregation analysis and Sanger sequencing), trio-WES provides an effective way to identify exon-level variants and determine the diagnosis of many rare monogenetic disorders, avoiding previous “diagnostic odysseys” experienced by many patients and their families (31–34). Nonetheless, we should note that technical limitations (exon-level sequencing only), along with the complexity of the genome (such as deep intronic or non-coding variants), may inevitably hinder the effectiveness of trio-WES. Whole-genome sequencing can offer advantages over trio-WES by identifying both exon-level and out-exon-level variants (such as structural, intronic and non-coding variants) but compared with trio-WES charges (almost $ 1,000 ~ 1,400 in China), the costs of whole-genome sequencing per subject in China is almost double than that of trio-WES. Due to the higher cost and more intense analysis, the application of whole-genome sequencing in clinical settings as a first-tier genetic diagnostic technique is still restricted (35–37). To date, trio-WES still provides an efficient and appropriate tool for first-tier genetic diagnosis worldwide and is invaluable for subsequent genetic counseling and relevant medical management. Therefore, it is meaningful to explore effective strategies to predict the diagnostic yield of trio-WES in clinical settings.

The diagnostic rate of trio-WES in clinical settings is likely influenced by a large variety of factors, such as the disorder type, phenotypic spectrum and age of onset (17, 36). For example, for individuals with congenital dermatological syndromes, the diagnostic rate can even reach 92% (17); perhaps the clear phenotypic presentation of those syndromes contributes to its high diagnostic rate. Thus, correct and precise assessment of subjects’ clinical data or phenotypic spectrum at the pre-WES stage is very important and presents a challenge, as it involves stringent collection and comprehensive analysis of the variable phenotypic features of every case. In the present study, we carefully and comprehensively collected detailed phenotypic information that can reflect neurodevelopment conditions objectively for every single subject to increase the diagnostic yield, and the overall diagnostic rates in the training, internal validation, and external validation cohorts were 50.3, 41.2 and 43.3%, respectively, which were higher than those reported in previous WES studies of patients with GDD/ID alone (27 to 39%) (8–10). Our findings concerning the diagnostic yield of using trio-WES for g-NDDs diagnosis further reinforce the conclusions presented by a research that the presence of GDD/ID along with multiple phenotypic features can enhance the diagnostic yield of trio-WES (2), emphasizing the importance of phenotypic feature enrichment in improving the exon-level variants detection rate of using trio-WES (18). According to the theory of the phenotype-to-genotype process and the phenotype-driven strategy, we speculate on the possibility that using key phenotypic factors that related to a high probability of having genetic variants detected by WES to construct a model for predicting trio-WES diagnostic rate, which may provide valuable information for personalized diagnostic regimens for g-NDDs children.

Among those four phenotypic factors (GDD/ID severity, NDC complexity, ASD, and HCA) identified in our study, the strongly top 3 associated features with positive trio-WES results were severe-profound GDD/ID (OR: 4.865, 95% CI: 2.213–10.694), followed by complicated NDC complexity (OR: 3.731, 95% CI: 1.399–9.950) and ASD (OR: 3.256, 95% CI: 1.479–7.168). The current findings suggest that severe-profound GDD/ID, ASD and a broad spectrum of NDCs may share genetic backgrounds in the context of rare monogenic NDDs, leading to a higher diagnostic rate of trio-WES. In the Simons Foundation Autism Research Initiative (SFARI, http://gene.sfari.org/) database, there are over one thousand genes involved in gene expression regulation and neuronal communication functions that are implicated in susceptibility to ASD and other coexisting NDCs, such as ADHD (38). Alterations in the functions of neuronal communication and gene expression regulation are also known to be closely associated with GDD/ID-related genes (39), which may explain the shared genetic backgrounds among severe-profound GDD/ID, ASD and a broad spectrum of NDCs. Moreover, we also revealed that HCA (OR: 2.788, 95% CI: 1.148–6.774) were moderately associated with positive trio-WES results under g-NDDs conditions. We speculate that in the process of craniofacial development, neuronal crosstalk and reciprocal signaling between the craniofacial ectoderm and neural crest cells play crucial roles in the regulation of craniofacial patterning and morphogenesis (40, 41). The process of neural crest development is regulated by epigenetic modifications, including chromatin remodeling, histone modification and DNA methylation (42). Alterations in gene expression regulation signaling and associated neuronal communication can result in disruptions in neural crest development, leading to a set of syndromes affecting a broad spectrum of congenital craniofacial malformations, among which HCA is prominent. Therefore, variants in genes involved in gene expression regulation and neuronal communication may impair multiple neurodevelopmental processes, causing severe-profound GDD/ID, ASD, and a broad spectrum of NDCs in addition to HCA. Our findings demonstrate that the presence of these four phenotypic features might indicate abnormalities in genes related to gene expression regulation or neuronal communication and may be strongly linked to positive trio-WES results, providing novel insights into the genotype–phenotype associations of g-NDDs. On the other hand, previous studies had already revealed that the four phenotypic features (severe-profound GDD/ID, having ASD, complicated NDC complexity, and HCA) are strongly established as signifiers of rare monogenic NDDs (2, 5, 43), and variants at exon-level had also been identified as the main cause of rare monogenic NDDs (44); given these close associations among them, it is reasonable to presume that a g-NDDs subject exhibiting more phenotypic signifiers related to rare monogenic NDDs may have more probabilities of harboring relevant exon-level variant(s), and thus can be diagnosed more easily via trio-WES. However, due to the lack of enough g-NDDs cases with confirmed variants outside exons (such as in introns), we cannot determine whether there have significant differences of those phenotypic signifiers between cases carrying variants at exon-level and at out-exon-level. More genotype–phenotype analyses are still needed to corroborate our speculation, and will be the focus of our future work.

The present study revealed that the established logistic regression model based on those four easily-obtained phenotypic factors exhibited good calibration and discrimination with high accuracy and precision in both the training and validation cohorts. However, the current model has several limitations. First, although the FNR of this model across training and validation sets (around 23% ~ 26%) was acceptable for a primary study, as many studies demonstrated that the predictive model’s FNR of being around 25% was tolerable especially for a preliminary study (45–47); it still showed relatively higher compared with the low FPR (around 12% ~ 18%) of the model, suggesting that the predictive model needs further refinement; perhaps it was too simplistic that all included indicators were binary variables, which can inevitably affect the model performance. Further improvements, such as introducing more polytomous variables or complicated variables into model, are required. Second, this was a pilot study, and the number of subjects enrolled in the current study was relatively small. Moreover, it is essential to establish a rigorous evaluation framework that ensures reproducibility across hospital and is independent of the individual physician’s judgment, which indicated that there have insufficient evidences to support this model could apply in the clinical practice directly; more multicenter studies (center number > 2) with a large sample size (case size > 500) and rigorous evaluation framework are needed to further validate our model. Moreover, our predictive model was based on a retrospective analysis; how it performs in prospective studies remains to be further evaluated.

## Conclusion

In conclusion, we found the potential linear relationship between trio-WES-diagnostic rates and the phenotypic enrichments in g-NDDs children for the first time, indicating the possibility of applying a logistic regression model based on phenotypic features to predict the personalized diagnostic rates of using trio-WES in children with g-NDDs. However, due to the false negatives existed in the established model of this pilot study, this model could not apply directly in the clinical practice as its current form; further improvements are required to reduce false negatives toward 0%.

## Data Availability

The original contributions presented in the study are included in the article/Supplementary material, further inquiries can be directed to the corresponding authors.

## Footnotes

^1^ http://www.ncbi.nlm.nih.gov/clinvar
